# The Prospective Association between Early Life Growth and Breast Density in Young Adult Women

**DOI:** 10.3390/cancers16132418

**Published:** 2024-06-30

**Authors:** Rachel Lloyd, Sarah Pirikahu, Jane Walter, Gemma Cadby, Nicole Warrington, Dilukshi Perera, Martha Hickey, Christobel Saunders, Michael Hackmann, David D. Sampson, John Shepherd, Lothar Lilge, Jennifer Stone

**Affiliations:** 1 Genetic Epidemiology Group, School of Population and Global Health, The University of Western Australia, Perth, WA 6009, Australia; rachel.peake@uwa.edu.au (R.L.);; 2 University Health Network, Toronto, ON M5G 2C4, Canadalothar.lilge@uhn.ca (L.L.); 3 Institute for Molecular Bioscience, The University of Queensland, Brisbane, QLD 4067, Australia; 4 The Frazer Institute, The University of Queensland, Woolloongabba, QLD 4102, Australia; 5 Department of Public Health and Nursing, K.G. Jebsen Center for Genetic Epidemiology, Norwegian University of Science and Technology, NO-7491 Trondheim, Norway; 6 Department of Obstetrics and Gynaecology, University of Melbourne and the Royal Women’s Hospital, Melbourne, VIC 3052, Australia; 7 Department of Surgery, The University of Melbourne, Parkville, VIC 3052, Australia; 8 School of Human Sciences, The University of Western Australia, Crawley, WA 6009, Australia; michael.hackmann@uwa.edu.au; 9 School of Computer Science and Electronic Engineering, The University of Surrey, Guildford, Surrey GU2 7XH, UK; 10 Epidemiology and Population Sciences in the Pacific Program, University of Hawaii Cancer Center, Honolulu, HI 96813, USA; 11 Medical Biophysics, University of Toronto, Toronto, ON M5G 1L7, Canada

**Keywords:** breast density, breast cancer risk, early life growth, optical breast spectroscopy, dual x-ray absorptiometry, the Raine Study

## Abstract

**Simple Summary:**

Breast cancer is the most common cancer worldwide and a leading cause of cancer-related deaths in women. Mammographic breast density, the relative amount of fibroglandular tissue as seen on a mammogram, is one of the strongest predictors of breast cancer risk, with higher breast density associated with greater risk. Breasts undergo extensive fibroglandular tissue proliferation in early life, making this a potential window of vulnerability for carcinogen exposures. Early-life growth (e.g., height and weight) is also associated with breast cancer risk. Research into the ‘developmental origins of health and disease’ paradigms investigate the pathways of risk for disease in later life from early-life exposures across developmental phases. However, little is known about the association between early-life growth and breast density. Further investigation into these risk pathways could increase knowledge of disease etiology and help identify women at increased risk at an earlier age and inform potential mediation of breast cancer risk.

**Abstract:**

Breast density is a strong intermediate endpoint to investigate the association between early-life exposures and breast cancer risk. This study investigates the association between early-life growth and breast density in young adult women measured using Optical Breast Spectroscopy (OBS) and Dual X-ray Absorptiometry (DXA). OBS measurements were obtained for 536 female Raine Cohort Study participants at ages 27–28, with 268 completing DXA measurements. Participants with three or more height and weight measurements from ages 8 to 22 were used to generate linear growth curves for height, weight and body mass index (BMI) using SITAR modelling. Three growth parameters (size, velocity and timing) were examined for association with breast density measures, adjusting for potential confounders. Women who reached their peak height rapidly (velocity) and later in adolescence (timing) had lower OBS-breast density. Overall, women who were taller (size) had higher OBS-breast density. For weight, women who grew quickly (velocity) and later in adolescence (timing) had higher absolute DXA-breast density. Overall, weight (size) was also inversely associated with absolute DXA-breast density, as was BMI. These findings provide new evidence that adolescent growth is associated with breast density measures in young adult women, suggesting potential mediation pathways for breast cancer risk in later life.

## 1. Introduction

Breast cancer is the most common cancer worldwide and a leading cause of cancer-related deaths in women [1]. Mammographic breast density, the relative amount of fibroglandular tissue as seen on a mammogram, is one of the strongest predictors of breast cancer risk, with higher breast density associated with greater risk [2,3]. Despite the large variation in breast density between women of similar age, breast density measures are highly correlated over time within women [4]. That is, if a woman has relatively high breast density for her age at 50 then it is likely she will have relatively high breast density for her age at 65 [4]. Large twin studies have estimated that genetic factors are responsible for approximately 60% of the variation in breast density between women [5,6], leaving 40% of the variation potentially attributable to environmental/lifestyle factors. Age and body mass index (BMI) are the strongest predictors of breast density and explain between 7 and 15% of this variation when combined with reproductive factors [7]. Together, these data suggest that breast density is established by young adulthood and tracks well through adult life, largely determined by genes. Reproductive factors like having children and going through menopause then act to, on average, decrease breast density as women age [4].

Research into the ‘developmental origins of health and disease’ (DOHaD) paradigms investigates the pathways of risk for disease in later life from early-life exposures across developmental phases [8,9]. Childhood and pubertal linear growth (e.g., height and weight) are associated with breast cancer risk. Tallness in childhood increases risk whilst an inverse association between early-life adiposity and breast cancer risk has been reported [10,11,12,13,14]. It is thought that the same hormonal and environmental factors that determine growth also influence breast tissue development. Breast density is an ideal intermediate endpoint to investigate the association between early-life exposures and risk of breast cancer because of its established associations with anthropometric measures [15,16,17,18], growth factors/hormones [15,19,20,21] and breast cancer risk. Breast density also tracks well over time within women from their adult years, with studies demonstrating this correlation from as early as age 30 [4,22].

Little is known about the association between early-life growth and breast density. Breasts undergo extensive fibroglandular tissue proliferation in early life, making this a potential window of vulnerability for carcinogen exposures [23,24,25]. Randomised controlled trials (RCT) suggest that the same sex steroid hormones and growth factors that regulate height and weight also modulate breast fibroglandular tissue proliferation and, potentially, breast cancer risk [20,21]. A 2014 review of epidemiologic studies investigating early-life body size and adult mammographic breast density found inconsistent results: two studies demonstrated a significant inverse association between body size in early life and breast density in premenopausal women aged 27 years (range 25–29 years) and 49 years (range 42–58 years), whereas three studies reported no association in premenopausal women [26]. However, several subsequent studies suggest that greater adiposity in childhood and adolescence is inversely associated with breast density measures (percent dense volume and absolute dense volume) in women throughout adult life [17,19,27,28]. A study by Denholm et al. showed that pubertal linear growth, particularly pubertal height growth, was positively associated with Magnetic Resonance Imaging (MRI) measured breast tissue composition (percent water) in young women but inversely associated with pubertal weight growth [15]. Two studies have investigated whether the association between childhood anthropometric measures and post-menopausal breast cancer risk is mediated by breast density [13,18]. Anderson et al. reported that adjustment for breast density attenuated the association between childhood body mass index (BMI) and risk of overall breast cancer but did not impact the risk associations with birthweight and childhood height. Rice et al. estimated that around 26% of the association between measures of childhood body size and post-menopausal breast cancer could be mediated by breast density. Further research is therefore needed to validate associations between early-life growth and breast density and inform potential mediation of breast cancer risk, particularly those with longitudinal growth data.

This study investigates the prospective association between early-life growth (the period of growth following the adiposity rebound which occurs between 3 and 7 years [29] until peak height and weight are reached following puberty) and breast density in a cohort of female participants aged 27–28 years within the Raine Study, one of the largest and most comprehensively characterised pregnancy cohorts internationally [30]. We examine associations between pubertal linear growth measures (size, timing and velocity) for height, weight and BMI measures collected throughout childhood and adolescence and measures of breast density. Breast density measures were collected using Optical Breast Spectroscopy (OBS) and Dual X-ray Absorptiometry (DXA), both shown to be highly correlated with mammographic breast density in screen-aged women [31,32,33]. Increased understanding of how early-life growth is associated with breast density throughout a lifetime could help define the ‘critical windows’ of modifiable environmental exposures which contribute to breast cancer risk.

## 2. Materials and Methods

### 2.1. Study Participants and Recruitment

The Raine Study is an ongoing prospective pregnancy cohort study established in 1989 with extensive data collection at multiple time points, including height and weight measures taken at ages 8, 10, 14, 17, 20 and 22. As described previously, female participants from the Raine Study were recruited into the current study at age 27 (Gen2-27, n = 452) and again at age 28 years (Gen2-28, n = 356) as part of the ongoing study recruitment by the Raine Study [34]. A flowchart of recruitment and exclusions can be found in Supplementary Materials Figure S1.

Study participation at ages 27 and 28 included completion of an extensive epidemiological questionnaire, measurement of height and weight, an OBS scan at both ages and both a whole-body and breast-DXA scan at age 28 (only). Gen2-27 participants were not invited to have a DXA scan.

### 2.2. Exclusion Criteria

Women previously diagnosed with breast cancer or who had undergone bilateral breast surgery (including mastectomy, lumpectomy, augmentation and reduction) were excluded (n = 2). Pregnant women were unable to undergo DXA scans due to low-level radiation exposure. Women who did not have height and weight measures for at least three ages between 8–22 years were excluded (n = 35).

### 2.3. Measuring Breast Density Using OBS

Participants were asked to undress from the waist up and change into an open-fronted hospital gown for the examination. Participants chose an appropriate breast size from four cups representing approximate bra cup sizes A–D. A trained research assistant performed a reference measure on a static silicone phantom mould using the chosen cup. Participants were then asked to place the cup over their left breast and hold it in place during the scan. The scan took up to five minutes, depending on breast size. The process was repeated for the right breast followed by a second reference measurement. A quality control check was performed immediately to determine whether a repeat scan was required.

Breast tissue composition measures using chromophore concentrations were calculated [35], providing measures of OBS%water, OBS%collagen and OBS%lipid. A combined measure of %water plus %collagen (henceforth, OBS%water + collagen) was also calculated. An average of the left and right breast was used for each participant.

### 2.4. Measuring Breast Density Using DXA

DXA scans of both breasts were carried out using a clinical GE DXA machine (GE Lunar iDXA Advance, Boston, MA, USA) according to the breast density measurement and calibration protocol previously outlined [32]. Briefly, participants were asked to remove jewellery and clothing from the waist up and change into an open-fronted hospital gown. Participants lay on their left side, positioning the left breast while holding the right breast out of frame during the scan before turning over to repeat on the opposite side. A repeat measure was done for the left breast. The scans took approximately two minutes per side.

The total projected breast area was manually delineated on each image, and the percent fibroglandular dense volume (%FGV) [36], absolute dense volume (FGV) and total breast volume were computed. Non-dense volume (NFGV) was calculated via subtraction. Scans containing artefacts were excluded from the analysis (n = 2). Repeated measurements of the left breast were compared, and those with differences in %FGV > 10% or FGV > 200 cm^3^ were re-checked for image quality. An average of the left and right breast was used for each participant.

### 2.5. Statistical Analyses

Descriptive statistics (counts and percentages for categorical variables or means and standard deviations for continuous variables) were used to summarise participant characteristics of OBS and DXA. Age at births of first and last child were centred on their mean, and all women having never given birth were assigned 0.

### 2.6. Growth Curve Analysis

**Figure 1:**
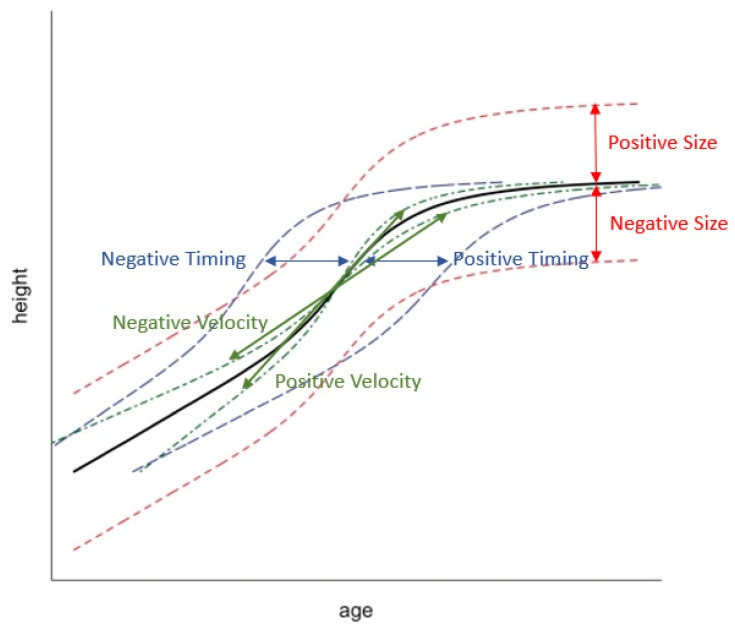
Illustration of the SITAR model for height in puberty taken from Cole et al., 2010 [37]. The solid line is the mean growth curve; the short dashed red lines indicate a vertical or height shift in the curve corresponding to the size parameter value, whereby a negative size value indicates size lower than the mean; the long dashed blue lines indicates a horizontal or age shift corresponding to the timing parameter value for age at peak growth velocity (APV) and indicates whether an individual reached their APV at an earlier or later (age) than average (negative timing means earlier than the mean) and the dot-dashed green lines represent a shrinking–stretching of the age scale reflecting velocity parameter value relating to the peak growth velocity (PV), which indicates whether an individual has a shorter or longer growth spurt compared with the population mean (negative velocity means a longer growth spurt than the mean).

Growth curve analysis was performed using superimposition by translation and rotation (SITAR) modelling [37]. Participant data with three or more height and weight measurements from ages 8, 10, 14, 17, 20 and 22 were used to generate 3-parameter growth curves for height, weight and BMI separately. The fitted growth curves enable individual estimation of growth size (cm/kg), timing (year) and velocity (fractional), by fitting these terms as random effects. The ‘size’ parameter value relates to a vertical shift in an individual’s curve from the mean population size and indicates whether an individual is smaller or larger than average (negative size indicates lower than the mean). The ‘timing’ parameter value relates to a horizontal shift for age at peak growth velocity (APV) and indicates whether an individual reached their APV earlier or later (age in years) than average (negative timing means earlier than the mean). The ‘velocity’ parameter value relates to the peak growth velocity (PV), which indicates whether an individual has a shorter or longer growth spurt compared with the population mean (negative velocity means a longer growth spurt than the mean). SITAR modelling for weight measurements performed better without the inclusion of the timing parameter in the random effects, which was tested for using the explained variance. Two models (size + timing, size + velocity) were generated with different random effect combinations for the weight variables. SITAR modelling for BMI measurements performed better without the inclusion of the timing parameter in any combination of random effects; parameter values were generated for size and velocity only. Figure 1 illustrates a SITAR model for height during puberty and demonstrates a visual representation of the shift in curves for size, timing and velocity.

### 2.7. Associations between Growth Curves and Breast Density Measures

Diagnostic plots of age-adjusted residuals were checked for the model assumption of normality for each of the breast density measures. This required %FGV, FGV and NFGV to be square root transformed. Multivariable linear regression was used to identify the best-fitting model to describe associations between each of the breast density measures and age, BMI, ethnicity (European/Caucasian, Asian, Other), oral conceptive use, reproductive history (i.e., parity, breastfeeding, contraceptive use, age of menarche), family history of breast cancer, breast skin colour (light, light/medium, medium, medium/dark, dark) and tobacco smoking; at least one cigarette per day for three months or longer (former, never, current) and alcohol consumption; at least one alcoholic drink a week for six months or longer (former, never, current). Backward stepwise regression was performed for the multivariable models using a cut-off *p*-value of <0.05. Model fit was compared using likelihood ratio tests and the Akaike Information Criterion. Age and BMI, established predictors of breast density, were included in all multivariable best-fitting models. Models for OBS%water + collagen and %FGV were also adjusted for cup size. Models for FGV were adjusted for cup size and breast skin colour.

The pubertal growth parameter values (size, timing and velocity) from each of the growth curve analyses for weight, height and BMI were added as covariates to each of the best-fitting models to examine their associations with OBS%water, OBS%water + collagen, OBS%lipid (for both the complete OBS dataset (n = 453) and the DXA subset (n = 253)), %FGV, FGV and NFGV. A fully adjusted multivariable model was also examined to assess sensitivity of the associations. All models were examined to ensure homoscedasticity. 

Approval to conduct this research was provided by the University of Western Australia in accordance with its ethics review and approval procedures (2020/ET000013).

## 3. Results

A total of 536 bilateral OBS scans were performed and of these, 490 participants received complete OBS data after processing, completed an epidemiological questionnaire and met the study eligibility requirements. Further exclusions due to missing data resulted in a final sample of 453 women with complete data, including height and weight measurements from three or more ages. Of these, a subset of 253 participants also had DXA-breast density measurements for analysis.

Table 1 provides characteristics for the 453 participants with complete OBS data and the subset of 253 women with DXA measures, separately. The characteristics between both groups were very similar. Overall, over 88% were of European/Caucasian ethnicity, had an average age of 27.3 years (sd = 0.9) at the 27-year follow-up and a BMI of 25.5 (kg/m^2^, sd = 6.1). Most had no live births (82.6%), had never breastfed (83.4%) and the mean age of menarche was 12.9 years (sd = 1.5). Most women had no family history of breast cancer (72.6%). The mean of OBS%water + collagen was 34.4% (sd = 10.7) and the mean of %FGV was 46.6% (sd = 17.8).

Table 2 provides the summary statistics for the growth parameter values (size, timing and velocity) from each of the growth curve analyses.

**Figure 2:**
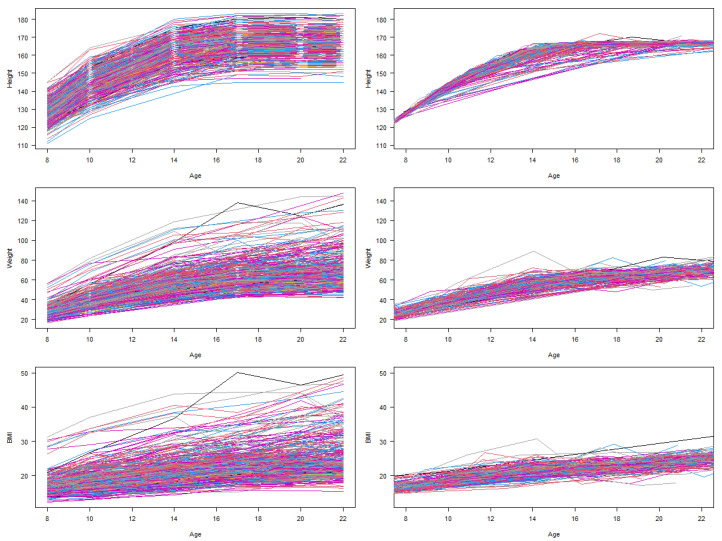
Plots showing the raw growth curve plots for height, weight and BMI (y-axis) against age (x-axis) on the left. The plots on the right show the fitted growth curves after the SITAR modelling (n = 453).

Figure 2 shows the raw growth curve plots for height, weight and BMI (y-axis) against age (x-axis) on the left. The plots on the right show the fitted growth curves after accounting for the size, timing and velocity parameters after SITAR modelling.

### 3.1. Height

From Table 3, both OBS%water and OBS%water + collagen were negatively associated with the timing of height growth, indicating a later age at peak growth velocity for women with lower OBS%water and OBS%water + collagen. For every year later of a woman’s peak growth velocity, their OBS%water and OBS%water + collagen decreased by 2.7% and 3%, respectively; that is, the later a woman reached her peak height, the lower their breast density as a young adult.

Both OBS%water and OBS%water + collagen were positively associated with the size of height growth. For every centimetre increase in a woman’s peak height, their OBS%water and OBS%water + collagen increased by 0.17% and 0.2%, respectively; that is, taller women have increased breast density in young adulthood.

Both OBS%water and OBS%water + collagen were negatively associated with the velocity of height growth, indicating that women who had a faster growth spurt starting at a later age have lower breast density in young adulthood compared to those that had a slower, longer growth spurt period starting at an earlier age.

Similar associations with the height parameters (size, timing or velocity) for OBS%water were also found for the reduced subset of women who also had DXA measures (n = 253) (see Supplementary Information Table S1).

Conversely, OBS%lipid was positively associated with the timing of height growth. For every year later for a woman’s peak growth velocity, their OBS%lipid increased by 3.5%, indicating a later age at peak growth velocity for women with higher percent “fatty” breast tissue. That is, the later a woman reached her peak height, the higher their percentage of breast fat in young adulthood. OBS%lipid was positively associated with the velocity of height growth indicating that women who have a faster growth spurt have a higher percentage of “fatty” breast content and therefore lower breast density compared to those that have a slower, longer growth spurt period, starting earlier in age.

No evidence of association with the height parameters (size, timing or velocity) was found for the DXA measures.

Similar associations with the height parameters (size, timing or velocity) for OBS%water, OBS%water + collagen and OBS%lipid were also found with a fully adjusted multivariable model (see Supplementary Information Table S2).

### 3.2. Weight

FGV and NFGV were both negatively associated with the size of weight increase, indicating that women with an increased early-life weight have both lower dense and non-dense breast tissue. FGV was positively associated with the velocity of weight increase, indicating that women whose early-life weight increased more rapidly, i.e., those with a faster growth spurt, have increased breast density as a young adult compared to those who had a slower, longer growth spurt period.

FGV was positively associated with the timing of weight growth, indicating a later age at peak growth velocity for women with higher breast density. That is, the later a woman reached her peak weight (maximum weight reached in adolescence), the higher their breast density as a young adult.

No evidence of association with the weight parameters (size, timing, or velocity) was found for the OBS measures.

### 3.3. BMI

FGV and NFGV were both negatively associated with the size of BMI, indicating that women with an increased early-life BMI have both lower dense (FGV) and non-dense (NFGV) breast tissue. No evidence of association between the BMI parameters (size or velocity) was noted for the OBS measures.

**Table 3:** Multivariable regression results for OBS (n = 453) and DXA (n = 253) breast density measures. Fibroglandular dense volume and non-dense volumes were square root transformed. ^1^ Models adjusted for Age and BMI and Cup Size ^2^ Models adjusted for Age, BMI ^3^ Models adjusted for Age, BMI, Cup Size and Breast Skin Colour ^4^ SITAR modelling using only size and velocity as fixed and random effects ^5^ SITAR modelling using only size and timing as fixed and random effects. The likelihood ratio test *p*-values for the effect of cup size and breast skin colour are all <0.01. Significant results shown in bold. Level of Significance: ≤0.001 ***; ≤0.01 **; ≤0.05 *; %FGV, Percent Fibroglandular Volume; FGV, Percent Fibroglandular Dense Volume; NFGV, Non-Dense Volume.

	OBS (β, 95% CI)			DXA (β, 95% CI)		
	%Water + Collagen ^1^	%Water ^2^	%Lipid ^2^	%FGV ^1^	FGV ^3^ (cm^3^)	NFGV ^1^ (cm^3^)
Height Size (cm)	0.20 (0.03, 0.38) *	0.17 (0.04, 0.31) **	−0.19 (−0.42, 0.03)	0.02 (0.00, 0.04)	0.03 (−0.05, 0.10)	−0.08 (−0.17, 0.00)
Height Timing (year)	−3.00 (−5.20, −0.68) **	−2.70 (−4.50, −0.92) **	3.50 (0.56, 6.40) **	−0.02 (−0.32, 0.28)	0.31 (−0.68, 1.30)	0.28 (−0.91,1.50)
Height Velocity	−23.00 (−38.0, −7.50) **	−21.00 (−33.0, −9.50) ***	27.00 (7.10, 46.00) **	−0.32 (−2.5, 1.90)	1.10 (−6.10, 8.40)	3.60 (−5.20, 12.00)
Weight Size ^4^ (kg)	0.11 ( −0.12, 0.35)	0.08 (−0.09, 0.26)	−0.11 (−0.40, 0.18)	0.00 (−0.02, 0.03)	−0.10 (−0.19, −0.02) **	−0.12 (−0.22, −0.02) **
Weight Velocity ^4^	−2.00 (−15.00, 11.00)	1.10(−9.00, 11.00)	4.90 (−12.00, 21.00)	1.10 (−0.39, 2.60)	5.90 (0.96, 11.00) *	0.33 (−5.60, 6.20)
Weight Timing ^5^ (year)	−0.36 (−2.70, 1.90)	−0.01 (−1.80, 1.80)	1.10 (−1.80, 4.00)	0.21 (−0.05, 0.48)	1.00 (0.18, 1.90) **	0.09 (−0.94, 1.10)
BMI Size	0.33 (−0.18, 0.84)	0.26 (−0.13, 0.65)	−0.28 (−0.92, 0.36)	0.01 (−0.05, 0.07)	−0.23 (−0.42, −0.04) **	−0.32 (−0.55, −0.09) **
BMI Velocity	−1.20 (−6.80, 4.50)	1.40 (−2.90, 5.80)	2.70 (−4.40, 9.80)	0.59 (−0.09, 1.30)	2.00 (−0.24, 4.20)	−0.74 (−3.40, 1.90)

## 4. Discussion

This study is the first to investigate the associations between early-life growth and breast density in young adult women using OBS and DXA to measure breast density.

**Figure 3:**
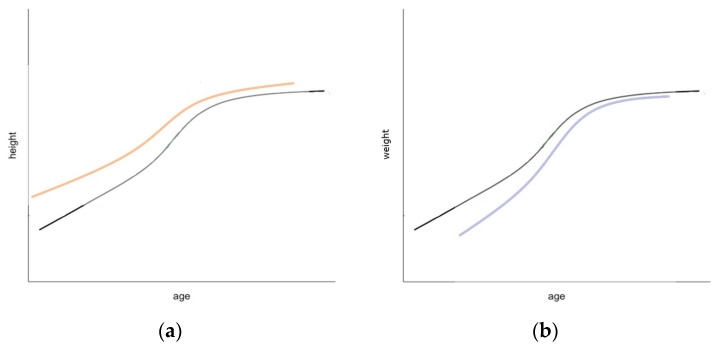
Descriptive curve (not to scale) of height growth during adolescence associated with higher breast density compared to the mean (**a**); descriptive curve (not to scale) of weight growth during adolescence associated with higher breast density compared to the mean (**b**).

We found that women who reached their peak height slower (velocity), i.e., had a longer growth spurt, but reached their peak height at an earlier age (timing) have increased OBS-breast density in young adulthood. Women who were taller (size) during puberty have higher OBS-breast density, consistent with height associations with breast cancer risk [11,38]. Figure 3a shows an approximate curve (not to scale) describing a likely height growth curve associated with higher breast density. In terms of weight, women who had a shorter growth spurt (velocity) but reached their peak weight at a later age (timing) have higher absolute DXA -breast density (FGV) as young adults. Women who had an overall lower weight (size) and BMI during adolescence, have higher absolute DXA-breast density (FGV) as young adults, consistent with weight associations with breast cancer risk [13,39]. Figure 3b shows an approximate curve (not to scale) describing a likely weight growth curve associated with higher breast density. These findings provide new evidence that early-life height and weight growth modify breast density in young adults, potentially mediating breast cancer risk in later life.

It is well-established that taller women are at greater risk of breast cancer [38], although the mechanisms underlying this are poorly understood. It is thought that growth hormones, particularly insulin-like growth factor-1 (IGF-1), which influences promotion of cell proliferation and bone growth, may play a role in determining height (and breast density) in premenopausal women, which in turn increases breast cancer risk [40,41]. One study has demonstrated that IGF-1 concentrations tracked significantly within individuals before and during pubertal height growth spurts. Longitudinal associations with IGF-1 have been reported for both peak height velocity and age at peak velocity, with higher concentrations of IGF-1 observed 6 months prior to the onset of breast development for girls with earlier age of breast development, longer duration of puberty and earlier age at peak velocity [42]. As IGF-1 has also been shown to be positively associated with breast density in premenopausal women [43], this is consistent with this study’s findings that women who experienced a longer growth spurt during adolescence and reached their peak height at an earlier age had higher breast density in young adulthood. Breast density is positively associated with height, confirmed by a recent large international study, utilising data from 22 countries, showing increased adult height associated with absolute mammographic dense area (cm^2^) [44]. Height has also been reported to be positively associated with MRI-measured breast density in young women aged 25–29 [45], and childhood height has been shown to be positively associated with breast cancer [11] and adult breast density in most studies [19,46] but not all [47]. Overall, the literature is consistent with this study’s findings that women who were taller during adolescence had higher OBS-breast density as young adults. Combined, these findings suggest that the positive association between adult height and breast cancer risk could in part be mediated by breast density; however, evidence of mediation has not been demonstrated thus far [13].

We observed a negative association between weight and BMI in early life and young adult breast density, but only for DXA-measured absolute FGV, not percent. Negative associations between adiposity and breast density measures are consistent with the previous literature [15,17,19,27,45,48,49], but these associations often depend on the metrics used (e.g., volume- vs area-based, percent vs absolute) [50,51,52,53]. Again, the biological pathways responsible for these associations are poorly understood. It has been hypothesised that the pathway through which greater childhood adiposity is associated with breast density could be through lower IGF-1 concentrations seen in heavier girls [15,19]. A recent study by Dorgan et al. investigating metabolites in childhood serum that possibly mediate the inverse association between adiposity in early life and young adult breast density (MRI-measured percent dense volume and absolute dense volume in women aged 25–29) identified two metabolites (X-16576 and X-24588) which supported the association. Whilst it is still unclear how these metabolites act to mediate the association, further investigations could lead to increased understanding of the biological pathways relating childhood adiposity, breast development and breast cancer risk. Increased adiposity across varying developmental stages influences breast cancer risk differently. In premenopausal women, increased BMI is protective against breast cancer, whereas postmenopausal women with increased BMI are at increased cancer risk [54]. Increased childhood and adolescence weight is inversely associated with breast cancer risk in later life [13,39], which is consistent with this study’s finding that weight during adolescence was inversely associated with FGV, as was BMI. Previous studies have also demonstrated that mammographic density could mediate the association of childhood BMI and breast cancer risk in premenopausal women [13,18,54]. We also found that women who grew quickly had higher DXA-measured absolute breast density (FGV), which is consistent with studies demonstrating increased breast cancer risk in those who experienced rapid adolescent growth (as measured by BMI) [23,39].

Another consideration of increased early-life adiposity is the effect it plays on the timing of puberty onset, caused by an increase in ovarian and adrenal hormones that bring about thelarche and menarche in females. These critical stages of pubertal development, also including pubertal tempo (the time between onset of thelarche and subsequent onset of menarche) appear to influence the amount of fibroglandular and adipose tissue that make up the breast composition, which then persists into adulthood [54]. Multiple studies have demonstrated that increased body adiposity during childhood results in earlier pubertal development [55,56,57,58]. Ghadge et al. summarised studies reporting associations between timing of puberty and mammographic density and found that most report a positive association between age at menarche and mammographic density, but not all studies. This positive association directly opposes that of the association between age of menarche and breast cancer risk, suggesting the association of age at menarche and breast cancer risk is not likely mediated through breast density [44]. Earlier age at thelarche was also found to be inversely associated with mammographic density; however, it has been suggested that the early onset of thelarche in girls with greater early-life adiposity is then compensated by a slower progression to menarche as a result of the different hormonal influences in heavier girls [19].

A recent study by Houghton et al. suggests that perhaps more important than the age of onset of thelarche and menarche is the pubertal tempo. They reported that a slower pubertal tempo (the longer the time period between thelarche and menarche) was associated with higher MRI-measured percent breast density in young women (aged 25–29 years) [59]. A positive association between breast cancer risk and pubertal tempo has also been reported [60], suggesting that it is the growth velocity (duration of pubertal growth spurt), rather than the timing, that affects breast tissue composition. Denholm et al. provides further evidence supporting this theory as their study also indicates that it is changes in growth velocity (duration of pubertal growth spurt), rather than the timing, that affects breast tissue composition [15]. The current study demonstrated associations with both velocity and timing, and with breast tissue composition (OBS%water, OBS%water + collagen and OBS%lipid), although the associations were much stronger for velocity.

Strengths of this study include its repeated prospective anthropometric measures obtained during early life from over 450 female participants within the Raine Study. OBS and DXA, although relatively uncommon methods for breast density assessment, provide unique opportunities to measure breast density in younger women when compared to other more common methods of density assessment, like mammography and MRI. Due to radiation exposure and low absolute risk, mammography is not recommended for women under the age of 40 years. MRI is costly and not a readily accessible resource for younger women to obtain routine breast density measurement. Whilst the correlation between the OBS- and DXA-breast density measures has been discussed previously [61,62], it should be noted that the two modalities are not necessarily measuring the same quantity. DXA provides image-based measurements of fibroglandular tissue, similar to mammography, whilst OBS estimates measures of breast tissue composition based on the light attenuation characteristics of water, fat, haemoglobin and collagen. It is therefore not entirely unexpected that the respective associations of OBS and DXA measures with the height and weight growth parameters did not replicate one another. It is possible that there are properties of breast density that are associated with height that are different from those associated with weight.

In terms of potential sources of bias, there were no statistically significant differences in the covariates between those participants who were excluded (n = 35) and our study sample (n = 453). Also, the outcomes were unlikely to be impacted by selection bias as participants were unaware of their breast density.

## 5. Conclusions

This study shows that women who were taller in adolescence had higher OBS-breast density as young adults, consistent with height-associated breast cancer risk. We also found women who had an overall lower weight and BMI during adolescence had higher absolute DXA-breast density, again consistent with weight associations with breast cancer risk. These findings provide new evidence that adolescent height and weight growth is associated with breast density in young adults, potentially mediating breast cancer risk in later life. Future research investigating the period of breast development during pubertal growth could help inform disease risk pathways and possible lifestyle-changing interventions to reduce the risk of breast cancer. There are currently limited risk-reducing strategies for breast cancer and identifying ways to reduce breast density, particularly by targeting younger women, could maximise prevention efforts. Whilst lifestyle factors like increased weight and alcohol use have been shown to be positively associated with breast density, there is limited evidence to demonstrate weight or alcohol interventions to reduce breast density. Thus far, only endocrine therapy has been shown to reduce breast density and thereby reduce breast cancer risk [63]. Using height and weight information in combination with breast density measures in young adult women could help identify women at increased risk who could potentially benefit from early entry into screening programs or risk-reducing strategies.

## 6. Patents

L.L. is co-holder of a patent related to the technology. However, no commercial contract is in place. All other authors declare no financial or commercial conflicts of interest.

## Figures and Tables

**Table 1:** Table of characteristics for the participants with OBS measures (N = 453) and the subset with DXA measures (N = 253). Abbreviations: sd, Standard Deviation; OBS, Optical Breast Spectroscopy; DXA, Dual X-Ray Absorptiometry; %FGV, Percent Fibroglandular Volume; FGV, Percent Fibroglandular Dense Volume; NFGV, Non-Dense Volume.

Characteristics	OBS Chromophore (N = 453)	DXA (N = 253)
Age at questionnaire (sd)	27.3 (0.9)	27.4 (0.7)
BMI (sd)	25.5 (6.1)	25.5 (6.1)
Ethnicity (%) European/Caucasian	400 (88.3)	228 (90.1)
Asian	37 (8.2)	19 (7.5)
Other	16 (3.5)	6 (2.4)
Ever been pregnant (%) Yes	129 (28.5)	65 (25.7)
No	322 (71.1)	186 (73.5)
Missing	2 (0.4)	2 (0.8)
Number of live births (%) 0	374 (82.6)	216 (85.4)
1	36 (7.9)	19 (7.5)
2	34 (7.5)	13 (5.1)
3 or more	9 (2.0)	5 (1.9)
Age at first birth (sd)	23.6 (3.0)	23.7 (3.2)
Age at last birth (sd)	25.4 (2.5)	25.3 (3.2)
Ever or currently breastfeeding (%) Never	378 (83.4)	216 (85.4)
Former	49 (10.8)	28 (11.1)
Current	24 (5.3)	7 (2.8)
Missing	2 (0.4)	2 (0.8)
Currently using any contraceptives (%) Yes	301 (66.4)	167 (66.0)
No	119 (26.3)	83 (32.8)
Missing	33 (7.3)	3 (1.2)
Contraceptive Type (%) Pill	146 (32.2)	80 (31.6)
IUD	37 (8.2)	28 (11.1)
Injection	33 (7.3)	17 (6.7)
Other	83 (18.3)	42 (16.6)
No Contraception	119 (26.3)	83 (32.8)
Not Stated	35 (7.7)	3 (1.2)
Age of Menarche (sd)	12.9 (1.5)	12.9 (1.5)
Missing	7	6
Family history of breast cancer (%) No history	329 (72.6)	187 (73.9)
1st Degree	23 (5.1)	16 (6.3)
2nd Degree	99 (21.9)	50 (19.8)
Missing	2 (0.4)	0 (0.0)
Benign Breast Disease—not removed (%) No	417 (92.1)	235 (92.9)
Yes—Not removed	34 (7.5)	17 (6.7)
Missing	2 (0.4)	1 (0.4)
Smoking Status (%) Never	225 (49.7)	139 (54.9)
Former	106 (23.4)	56 (22.1)
Current	53 (11.7)	21 (8.3)
Missing	69 (15.2)	37 (14.6)
Alcohol consumption (%) Never	35 (7.7)	20 (7.9)
Ever	350 (77.3)	197 (77.9)
Missing	68 (15.0)	36 (14.2)
OBS Cup Size (%) 1 (A)	149 (32.9)	85 (33.6)
2 (B)	113 (24.9)	63 (24.9)
3 (C)	129 (28.5)	74 (29.2)
4 (D)	62 (13.7)	31 (12.3)
Measurement: (sd) OBS-%water	15.9 (8.1)	14..6 (7.7)
OBS-%lipid	50.7 (13.4)	53.4 (13.2)
OBS-%collagen	18.4 (7.1)	18.1 (7.4)
OBS-%water + collagen	34.4 (10.7)	32.7 (10.2)
DXA %FGV		46.6 (17.8)
DXA FGV (cm^3^)		240.0 (112.7)
DXA NFGV (cm^3^)		350.8 (291.6)

**Table 2:** Summary statistics for the growth parameter values produced via SITAR modelling (n = 453). Abbreviations: sd, Standard Deviation; Min, Minimum value; Max, Maximum Value.

SITAR Model Growth Parameters	Height Min, Max (sd)	Weight Min, Max (sd)	BMI Min, Max (sd)
Size (cm)	−21.5, 16.1 (6.3)	−20.6, 65.9 (13.0)	−7.1, 18.0 (4.0)
Timing (years)	−2.6, 3.1 (0.9)	−2.3, 7.7 (1.6)	
Velocity (fractional)	−0.4, 0.5 (0.1)	−0.4, 1.0 (0.2)	−0.6, 1.7 (0.3)

## Data Availability

The data supporting this study’s findings are available on request from the corresponding author. The data are not publicly available due to privacy or ethical restrictions.

## Supplementary Materials

The following supporting information can be downloaded at: https://www.mdpi.com/article/10.3390/cancers16132418/s1, Table S1: Multivariable regression results for OBS-breast density measures for the subset with DXA measures (n = 253); Table S2: Multivariable regression results for OBS (n = 453) and DXA (n = 253) breast density measures; Figure S1: Flowchart of recruitment and exclusions.
