# Supplementary material for: The Prospective Association between Early Life Growth and Breast Density in Young Adult Women
Source: Cancers (Basel). 2024 Jun 30;16(13):2418. doi: 10.3390/cancers16132418 (PMC11240569; doi:10.3390/cancers16132418)
Supplement: Supplementary file 1 [file cancers-16-02418-s001.zip › cancers-3017336-supplementary.pdf]

Supplementary Information

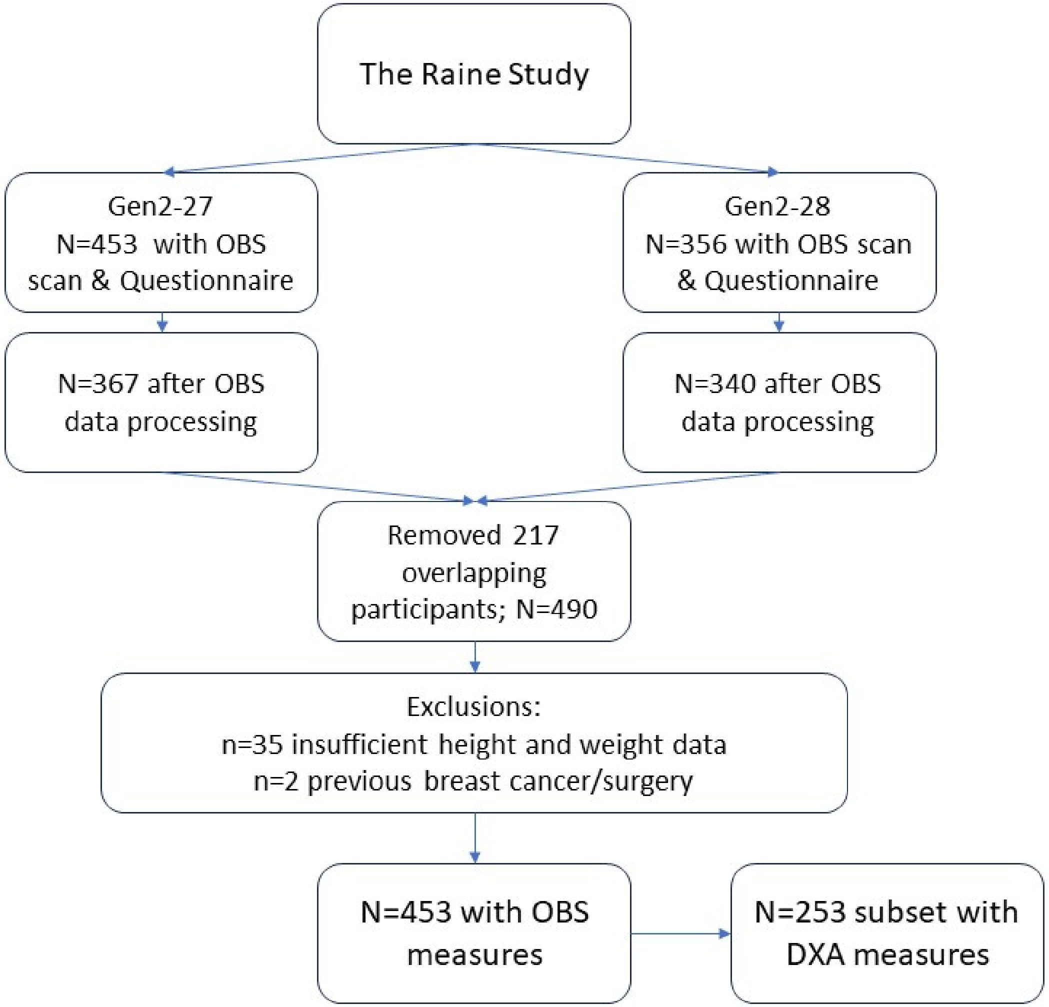

Figure S1. Flowchart of recruitment and exclusions.

Table S1. Multivariable regression results for OBS breast density measures for the subset with DXA measures (n=253). [1]Models adjusted for Age and BMI and Cup Size [2] Models adjusted for Age, BMI [3] SITAR modelling using only size and velocity as fixed and random effects [4] SITAR modelling using only size and timing as fixed and random effects. The Likelihood ratio test p–values for the effect of cup size and breast skin colour are all <0.01 . Signif. codes: <= 0.01 **; Beta indicates the change in OBS measure for each unit change in the sitar parameters.

	OBS (β, 95% CI)		
	%Water+Collagen[1]	%Water[2]	%Lipid[2]
Height Size (cm)	0.17 (−0.07,0.41)	**0.23 (0.05,0.42)****	−0.17 (−0.48, 0.15)
Height Timing (year)	−1.90 (−5.10,1.40)	**−3.00(−5.40,−0.49)****	2.00 (−2.20, 6.20)
Height Velocity	−18.00 (−42.0, 5.80)	**−27.0 (−45.0, −8.6)****	19.0 (−12.0,50.0)
Weight Size[3] (kg)	0.02 (−0.26, 0.31)	0.05(−0.16,0.27)	−0.04 (−0.40,0.33)
Weight Velocity[3]	4.40(−12.0,21.0)	4.60 (−8.00,17.0)	−0.92 (−22.0,20.0)
Weight Timing[4] (year)	0.80 (−2.10,3.70)	0.73 (−1.50,2.90)	−0.13 (−3.90,3.60)
BMI Size	0.26 (−0.38, 0.90)	0.24 (−0.24,0.72)	−0.21 (−1.00, 0.59)
BMI Velocity	0.43 (−7.0,7.9)	2.40 (−3.20,7.90)	1.30 (−8.10,11.0)

Table S2. Multivariable regression results for OBS (n=453) and DXA (n=253) breast density measures. Fibroglandular dense volume and non−dense volumes were square root transformed. Models fully adjusted for Age, BMI, Ethnicity, Age at menarche, Number of live births, Parity, Age of first birth, Age of last birth, Breastfeeding status, Benign breast disease, Breast skin colour, Family History, Smoking Status, Alcohol status, Contraception, Menstrual cycle week, Areolar size and Cup Size. [1] SITAR modelling using only size and velocity as fixed and random effects [2] SITAR modelling using only size and timing as fixed and random effects. Signif. codes: <= 0.001 *** ; <= 0.01 **; <= 0.05* ; %FGV, Percent Fibroglandular; FGV, Percent Fibroglandular Dense Volume; NFGV, Non−Dense Volume. Beta indicates the change in OBS/DXA measure for each unit change in the sitar parameters.

	OBS (β, 95% CI)			DXA (β, 95% CI)		
	%Water+Collagen	%Water	%Lipid	%FGV	FGV (cm^3)	NFGV (cm^3)
Height Size (cm)	**0.24 (0.05, 0.44)****	**0.19 (0.04, 0.34)****	**−0.29 (−0.51,−0.06)****	0.01 (−0.01, 0.04)	0.02 (−0.06, 0.10)	−0.04 (−0.14, 0.06)
Height Timing (year)	**−2.5 (−5.00, 0.05)***	**−2.40 (−4.40, −0.45)****	**3.10 (0.16, 6.00)***	0.01 (−0.34, 0.35)	0.09 (−1.00, 1.20)	0.07 (−1.30,1.40)
Height Velocity	**−20.00 (−37.0, −3.7)****	**−20.00 (−33.0, −6.60)*****	**25.00 (5.30, 44.00)****	0.30(−2.1, 2.80)	0.90 (−7.10, 8.90)	0.44 (−9.20, 10.00)
Weight Size[1] (kg)	0.09 (−0.16, 0.34)	0.05 (−0.15,0.25)	−0.11 (−0.41, 0.18)	0.00 (−0.03, 0.03)	−0.08 (−0.18, 0.03)	−0.11 (−0.23, 0.01)
Weight Velocity[1]	2.00 (−13.00, 17.00)	4.30(−7.20, 16.00)	3.50 (−13.00, 20.00)	1.10 (−0.72, 2.90)	3.70 (−2.4, 9.70)	−0.36 (−7.40, 6.70)
Weight Timing[2] (year)	0.07 (−2.50, 2.60)	0.40 (−1.60,2.40)	0.98 (−2.00, 3.90)	0.19 (−0.13, 0.51)	0.68 (−0.37, 1.70)	0.09 (−1.10, 1.30)
BMI Size	0.38 (−0.16, 0.92)	0.25 (−0.17, 0.68)	−0.22 (−0.85, 0.41)	0.03 (−0.04, 0.09)	−0.18 (−0.40, 0.03)	**−0.37 (−0.62, −0.12)****
BMI Velocity	−0.47 (−6.50, 5.60)	2.10 (−2.70, 6.80)	2.30 (−4.70, 9.30)	0.52 (−0.24, 1.30)	1.20 (−1.30, 3.70)	−0.95 (−3.80, 1.90)
